# The Association between Disturbed Eating Behavior and Socioeconomic Status: The Online Korean Adolescent Panel Survey (OnKAPS)

**DOI:** 10.1371/journal.pone.0057880

**Published:** 2013-03-05

**Authors:** Hae-Jeung Lee, Sangshin Park, Cho-il Kim, Doo-won Choi, Jung Sun Lee, Sun Min Oh, Eunyoung Cho, Hye Kyung Park, Kwang-il Kwon, Sang Woo Oh

**Affiliations:** 1 Channing Laboratory, Department of Medicine, Brigham and Women’s Hospital, Harvard Medical School, Boston, Massachusetts, United States of America; 2 Department of Nutrition, Harvard School of Public Health, Boston, Massachusetts, United States of America; 3 Center for Nutrition Policy and Promotion, Korea Health Industry Development Institute (KHIDI), Osong, Chungcheongbuk-do, Korea; 4 Department of Veterinary Integrative Biosciences, College of Veterinary Medicine and Biomedical Sciences, Texas A&M University, College Station, Texas, United States of America; 5 Embrain Co.Ltd, Seoul, Korea; 6 Department of Foods and Nutrition, University of Georgia, Athens, Georgia, United States of America; 7 Department of Preventive Medicine, Yonsei University College of Medicine, Seoul, Korea; 8 Nutrition Policy Division, Korea Food and Drug Administration, Osong, Chungcheongbuk-do, Korea; 9 Department of Family Medicine, Center for Obesity, Metabolism and Nutrition Dongguk University Ilsan Hospital, Goyang-Si, Gyeonggi-Do, Korea; Pennington Biomedical Research Center, United States of America

## Abstract

**Background:**

A limited amount of research, primarily conducted in Western countries, has suggested that higher socioeconomic status (SES) is associated with higher risk of eating disorders (EDs). However, little is known about this association in Asian countries. We examined the association of SES with disturbed eating behavior (DEB) and related factors in Korean adolescents.

**Subjects:**

A nationwide online panel survey was conducted in a sample of adolescents (*n* = 6,943, 49.9% girls). DEB was measured with the 26-item Eating Attitudes Test (EAT-26). Participants who scored ≥20 on the EAT-26 were considered to have DEB. Participants’ SES was determined based on self-reported household economic status.

**Results:**

The prevalence of DEB was 12.7%: 10.5% among boys and 14.8% among girls. Both boys and girls with DEB were more likely to perceive themselves as obese, experience higher levels of stress, and have lower academic achievement. The risk for DEB was significantly higher in boys of higher SES than in those of middle SES (OR = 1.45, 95%CI = 1.05–1.99 for high SES; OR = 5.16, 95%CI: 3.50–7.61 for highest SES). Among girls, higher risk of DEB was associated with the highest and lowest SES (OR = 1.52, 95%CI: 1.13–2.06 for lowest SES; OR = 2.22, 95%CI: 1.34–3.68 for highest SES).

**Conclusions:**

Despite the lower prevalence of obesity in Korea compared with Western countries, the prevalence of DEB in Korean adolescents was high, especially among girls. Moreover, the association between SES and DEB followed a U-shaped curve for girls and a J-shaped curve for boys.

## Introduction

Eating disorders (EDs) such as anorexia nervosa, bulimia nervosa, and binge ED are serious and growing health problems among adolescents [1]. Although a wide range of estimates of the prevalence of EDs in adolescents (3.2%–26.0%) has been reported based on studies using different methods in different settings and in different countries [2]–[9], it is clear that the incidence and prevalence of EDs in adolescents has been on the increasing during recent decades [10], [11]. Thus, it is critical that we better understand and identify adolescents at increased risk for EDs for purposes of early detection and appropriate management.

EDs are a multifaceted problem associated with various interacting factors related to adolescence, including biological, psychological, familial, and sociocultural variables. As risk factors in a review, perceived body image is strong risk factor and next being overweight is a well-known factor, and screen time including media and internet use were associated with the risk of eating disorders [12]. Residential region [13] and physical activity [14] may also affect the development of eating disorders. Among these factors, the association between socioeconomic status (SES) and EDs is of particular interest given that EDs have been shown to be more prevalent in white females of higher SES in Western countries [15]–[17]. Indeed, several studies have reported such trends not only among adolescent girls but also among adolescent boys; EDs were more prevalent in upper-middle-SES adolescents than in lower-SES adolescents [18]–[20]. However, these studies are limited in that they often failed to adequately sample from lower SES groups [21]–[24] even though higher rates of EDs have been observed in girls with lower SES than in their counterparts with higher SES [25]. Recent large community- or population-based studies have reported inconsistent results [18], [26].

Contemporary South Korean culture can be characterized as dynamic, with rapid growth and diversification in areas such as industrialization, urbanization, and economics. Concurrently the prevalence of obesity is increasing, especially with regard to adolescents. Based on Korea National Health and Nutrition Examination data the ratio of Korean adolescents suffering from obesity (BMI of 25 or higher) increased to 11.3% in 2009 up from 5.8% in 1997 [27]. This increase is significant and worrying even though the prevalence of obesity remains lower in Korea than other western countries. These changes clearly warn of the likelihood of an increasing prevalence of eating disorders in Korea. Other Asian countries will likely face these situations in the near future as they too embark on further development and globalization. Therefore the results of this research may suggest public health policy directions, further research and fields of enquiry well beyond Korea’s national boundaries.

Our understanding of the epidemiology of EDs and its associations with SES among adolescents in Asian countries is very limited [2], [3]. Causal conditions, if (partially) socio-culturally based are also likely to be both more similar between Asia and Korea, and more divergent between Western countries and Korea. The purpose of this study was to estimate the prevalence of disturbed eating behavior (DEB) in adolescents using a nationally nationwide sample of Korean adolescents and to examine associations between SES and DEB.

## Materials and Methods

### Sample

This study used cross-sectional baseline data from the Online Korean Adolescent Panel Survey (OnKAPS). The purpose of OnKAPS was to estimate the number of adolescents at risk for EDs and to identify the risk factors associated with these disorders. The study was initiated in 2009 and follow-up surveys were conducted every 2 years. A nationwide Korean adolescent panel was selected according to the distribution of this population across the 16 regions, adolescent age groups, and genders reported in the 2005 Korean Population Census. Our Candidate panels were members of Embrain Research Co. Potential respondents were invited to take part in the survey, which they could access upon agreement to participate. Of the 16,090 Korean students who received an invitation email, 9,327 contacted the survey webpage. A total of 7,711 individuals completed all survey questions. The sample used for analysis included 6,943 students aged 12–18 years (3,476 boys and 3,467 girls). We excluded 768 students who 1) did not match with basic information such as age or region, or 2) answered with “I don’t know” for our interesting variables in this study, or 3) recorded an extreme duration of web-survey (less than 5 minutes, over than 50 minutes). The response rate was 48%.

This survey was conducted by Embrain Online Research Company (Embrain Co., Ltd, Seoul, Korea) between October and November 2009. Use of this on-line research design was made possible by the 100% rate of Internet adoption in Korea [28]. This study was based on baseline data from the OnKAPS.

### Ethics Statement

This survey was approved by the Korea Food and Drug Administration Institutional Review Board, and all participants and their mother or father provided written informed consent before participating. All informed consent was obtained by internet or email.

### OnKAPS Questionnaires

OnKAPS questionnaires included questions addressing three domains: 1) socio-demographic characteristics (e.g., age, gender, height, weight, school type, economic status, and academic achievement); 2) eating disturbances and weight perceptions; and 3) dietary habits, physical activity, screen time (TV, video games, Internet), and stress level.

The questionnaire was designed to be answered in 15–20 minutes to ensure high-quality responses. This timeframe is suggested in order to avoid survey fatigue and maintain respondent attention on survey items [29]. The mean survey time was 16.0 minutes, and the questionnaire had been pilot-tested and revised to clarify the meanings of questions.

### Eating Attitudes Test-26 (EAT-26)

The Eating Attitudes Test-26 (EAT-26) is the most frequently used tool for identifying adolescents with DEB in a community setting [30], [31]. The EAT-26 was originally developed in the USA and based on American English. It has been translated into many languages. A validated Korean version of the EAT-26 for adolescents was used in this study [31]. Based on the results of the pilot test, the phrasing and literal translation of 4 questions seemed to be problematic and unclear in our online survey. Therefore we added an explanation with parenthesis to the Korean version of the EAT-26 to clarify the meaning of the following four items: “Display self-control around food,” “Give too much time and thought to food,” “Like my stomach to be empty,” and “Enjoy trying new rich foods”. The EAT-26 incorporates 26 items in a 4-point Likert scale, where the participants indicate the extent of their agreement or disagreement. Answers range from 3: always, 2: usually, 1: often, 0: sometimes/rarely to never (e.g.: ‘I engage in dieting behavior’). The responses on the 26 items are summed at the end and a total score, ranging from 0 (minimum) to 78 (maximum), is extracted. DEB was defined as scores of 20 or more on the EAT-26, which has been shown to be associated with abnormal eating attitudes and behavior [30]–[32]. Cronbach’s alpha of EAT-26 in our sample was 0.85.

### Socioeconomic Status

SES was determined based on responses to the question, “What is your household’s economic status?” The response categories were highest, high, middle, low, and lowest. To test the reliability of reported SES, we randomly selected 50 participants and asked their parents about their annual household income via phone. We found a significant correlation (*r* = 0.64, *p*<0.0001) between reported SES and annual income (Supplementary table1).

### Anthropometry

Participants’ height and weight and the size of their school-uniform pants were assessed. Body mass index (BMI = weight (kg)/squared height (m2)) was calculated based on self-reported weight and height. Weight status was defined according to data from the 2007 Korean National Growth Charts on BMIs by age (in months) and gender: a) underweight (BMI <5th percentile); b) normal (5th percentile ≤ BMI <85th percentile); or c) overweight (BMI ≥85th percentile) [33].

### Other Characteristics

Data on age, gender, school type, academic achievement, perceived economic status, stress level, perceived body weight, physical activity, and screen times were collected. The questions were as follows:

“What type of school and class do you attend?”; the response categories were single-gender school, single-gender class in coeducational school, or coeducational class in coeducational school. “How much stress do you usually feel?”; the response categories were highest, high, middle, low, and lowest. “What do you think your weight status is?”; the response categories were very thin, thin, normal, fat, and very fat. “Do you exercise three or more times per week and for 30minutes or more per session?”; the response categories were yes or no. “How often do you devote more than 2 hours per day to screen time (e.g., watching TV, playing video games, connecting to the Internet); the response categories were usually, sometimes, and never. For purposes of analysis, we collapsed the five levels of stress and academic achievement (highest, high, middle, low, and lowest) into three (high, middle, and low). The perceived weight status response data was also collapsed into 3 groups during analysis.

### Statistical Analysis

Due to the significant interaction between gender and SES (*P* for interaction <0.001), analyses were stratified by gender. Descriptive statistics were used to summarize the characteristics of the study sample. Numerical data was presented as mean values and standard error (SE). Proportion and SE of proportion was calculated for categorical variables. To compare the characteristics of Korean adolescents with and without DEB, *t*-tests and *chi*-square tests were performed for continuous and categorical variables, respectively.

Logistic regression models were used to examine the association between SES and DEB. Obesity, perceived weight status, screen time, stress, and academic achievement was significantly correlated with eating disturbance (P<0.05). Residential region (i.e. metropolitan, rural) could be associated with the risk of DEB [13]. Physical activity could affect development of eating disorder [14]. Multivariate model (MV) 1 adjusted for demographic and social factors: region (metropolitan area, middle–small city, rural area), school type (coeducation bi-gender classroom, coeducation single gender classroom, single gender school), BMI (underweight, normal, overweight), academic achievement (high, middle, low), stress level (high, middle, low), regular physical activity(≥30 min, 3days or more/week) (yes, no), and screen time in usual days (usually, sometimes, never). MV2 additionally was adjusted for perceived weight status in MV1 to observe the effect of this factor.

The prevalence (%) and 95% Confidence Interval of DEB were calculated according to SES by gender. Additionally, the proportion (%) and 95% Confidence Interval of those who were simultaneously overweight, very stressed, perceived themselves as fat, and had high academic achievement were calculated according to SES by gender, respectively. All analyses were performed using SAS 9.2 (Cary, NC). P-values less than 0.05 (two-tailed test) were considered to indicate statistical significance.

## Results

The prevalence of DEB in this national sample of Korean adolescents was 12.7% (95%CI: 11.9–13.4), and it was higher among Korean adolescent girls than boys (14.8% (95%CI: 13.6–16.0) vs. 10.5% (95%CI: 9.5–11.5)).

Table 1 shows the characteristics of the study subjects by gender and DEB. Boys with DEB were more likely to be overweight, attend a coeducational or single-gender class, perceive themselves as fat, experience high levels of stress, have lower academic achievement, and report less screen time. Girls with DEB were more likely to be taller and heavier, perceive themselves as fat, experience high levels of stress, and have lower academic achievement.

**Table 1:** Characteristics of boys and girls with or without Disturbed Eating Behavior (DEB).

	Boys			Girls		
	No-DEB	DEB	p	No-DEB	DEB	p
	(n = 3,111)	(n = 365)	value	(n = 2,954)	(n = 513)	value
Age (year) (mean±SE)	15.3±0.03	15.2±0.09	0.28	15.2±0.03	15.4±0.07	0.002
Anthropometric variables (mean±SE)						
Height (cm)	171.3±0.12	172.0±0.36	0.06	160.2±0.10	160.9±0.22	0.01
Weight (kg)	61.7±0.21	63.0±0.75	0.10	52.3±0.16	53.2±0.36	0.02
BodyMass Index(kg/m^2^)	20.9±0.06	21.2±0.22	0.29	20.3±0.05	20.5±0.12	0.14
Demographic variables						
School^1^, % (SE)						
Middle	89.8(0.7)	10.2(0.7)	0.53	87.0(0.8)	13.0(0.8)	0.002
High	89.2(0.8)	10.8(0.8)		83.4(0.9)	16.6(0.9)	
Urban or rural, % (SE)						
Metropolitan	57.1(0.9)	59.5(2.5)	0.66	57.1(0.9)	58.1(2.1)	0.38
Middle, small city	33.9(8.5)	32.6(2.4)		34.8(0.9)	32.4(2.0)	
Rural	9.0(0.5)	7.9(1.4)		8.1(0.5)	9.5(1.3)	
School type, % (SE)						
Coeducation- both gender classroom	33.4(0.9)	39.4(2.6)	0.05	39.3(0.9)	42.1(2.2)	0.49
Coeducation-single gender classroom	48.2(0.9)	45.5(2.6)		42.9(0.9)	40.7(2.2)	
Single gender school	18.4(0.7)	15.1(1.9)		17.8(0.7)	17.2(1.7)	
Obesity^2^, %(SE)						
Underweight	6.5(0.4)	12.9(1.8)	<.0001	6.3(0.5)	5.5(10)	0.60
Normal	80.5(0.7)	68.0(2.4)		82.4(0.7)	82.1(1.7)	
Overweight	13(0.6)	19.2(2.1)		11.3(0.6)	12.5(1.5)	
Perceived weight status, %,(SE)						
Thin	38.5(0.9)	40.5(2.6)	<.0001	20.3(0.7)	16.8(1.7)	0.0002
Normal	33.6(0.9)	22.3(2.2)		38.5(0.9)	32.2(2.1)	
Fat	27.9(0.8)	37.2(2.5)		41.2(0.9)	51.0(2.2)	
Lifestyle variables						
Screentime> = 2 hr/day, %(SE)						
Usually	16.5(0.7)	9.2(1.5)	0.001	12.2(0.6)	11.4(1.4)	0.08
Sometimes	21.0(0.7)	20.9(2.1)		17.4(0.7)	13.7(1.5)	
Never	62.5(0.9)	69.8(2.4)		70.4(0.8)	74.9(1.9)	
Regular physical activity (> = 30 min,3days and more/week), %(SE)	36.5(0.9)	40.6(2.6)	0.12	16.8(0.7)	18.5(1.7)	0.34
Stress, % (SE)						
High	42.3(0.9)	54.0(2.6)	<.0001	60.8(0.9)	79.3(1.8)	<.0001
Middle	40.3(0.9)	28.2(2.4)		30.7(0.9)	14.1(1.5)	
Low	17.4(0.7)	17.8(2.0)		8.5(0.5)	6.6(1.1)	
Education variables						
Academic achievements,% (SE)						
High	46.6(0.9)	38.4(2.6)	0.001	44.4(0.9)	36.5(2.1)	0.0008
Middle	26.2(0.8)	26.0(2.3)		25.6(0.8)	25.1(1.9)	
Low	27.2(0.8)	35.6(2.5)		30.0(0.8)	37.6(2.1)	

1 Sum of the value of row by DEB and non-DEB is 100%.

2 Underweight (BMI <5th percentile), normal (5th percentile ≤ BMI <85th percentile), overweight (BMI≥85th percentile) by in 2007 Korean National Growth Chart.

SE = Standard Error.

**Figure 1:**
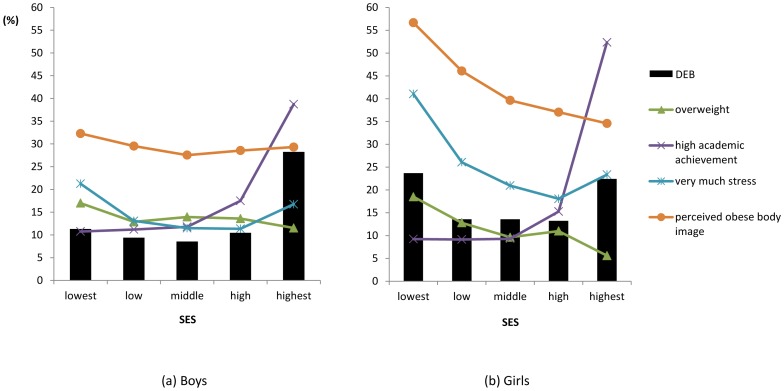
Distribution of Disturbed Eating Behavior (DEB) and related factors by Socioeconomic Status (SES) in boys and girls. We calculated the prevalence (%) and 95% Confidence Interval of DEB among those who were overweight, were very stressed, perceived themselves as fat, and had high academic achievement according to SES by gender.

We found an inverse association between SES and obesity. The prevalence of overweight individuals was highest in the lowest SES group and lowest in the highest SES group. By contrast, the proportion of individuals with high levels of academic achievement was highest among the highest SES group and lowest among the lowest SES group. The distribution of these factors by SES was similar for both genders, but the pattern was stronger in girls than in boys (Fig. 1). The proportion of overweight individuals, those who perceived themselves as fat, and those with high levels of stress also seemed to be highest in the lowest SES group in both genders. The prevalence of DEB was highest in the highest SES group, followed by the lowest SES group. The distribution of DEB across the five SES groups differed by gender: it was U-shaped in girls and J-shaped in boys (Fig. 1). We found a significant association between SES and DEB in the study sample (Table 2). Boys in the high or highest SES groups were at higher risk for DEB than were those in the middle SES group after adjusting for potential confounders (high vs. middle SES: OR = 1.45, 95%CI = 1.05–1.99; highest vs. middle SES: OR = 5.16, 95% CI = 3.50–7.61). On the other hand, girls in the lowest and highest SES groups had a higher risk of DEB than did those in the middle SES group (lowest vs. middle SES: OR = 1.52, 95%CI = 1.13–2.06; highest vs. middle: OR = 2.22, 95% CI = 1.34–3.68 in MV2).

**Table 2:** Associations between Socioeconomic Status and Disturbed Eating Behavior (DEB) in Korean adolescents by gender.

Boys	household economic status	No. of DEB/No of participants (%)	Age-adjusted	Multivariate model 1	Multivariate model 2
	Lowest	40/351 (11.4%)	1.91 (1.43–2.55)	1.13 (0.77–1.68)	1.06 (0.71–1.57)
	Low	89/945 (9.4%)	1.11 (0.83–1.49)	1.04 (0.78–1.39)	0.98 (0.73–1.32)
	Middle	112/1325 (8.5%)	1.00	1.00	1.00
	High	70/664 (10.5%)	1.30 (0.95–1.78)	1.39 (1.01–1.91)	1.45 (1.05–1.99)
	Highest	54/191 (28.3%)	4.33 (2.99–6.27)	5.05 (3.45–7.38)	5.16 (3.50–7.61)
	*P for trend*		*<.0001*	*<.0001*	*0.0001*
Girls	household economic status	No. of DEB/No of participants (%)	Age-adjusted	Multivariate model 1	Multivariate model 2
	Lowest	80/339 (23.6%)	1.98 (1.43–2.55)	1.59 (1.18–2.15)	1.52 (1.13–2.06)
	Low	136/1006 (13.5%)	0.98 (0.78–1.24)	0.90 (0.71–1.14)	0.87 (0.69–1.11)
	Middle	203/1485 (13.7%)	1.00	1.00	1.00
	High	71/534 (13.3%)	0.99 (0.74–1.32)	1.04 (0.78–1.40)	1.06 (0.79–1.42)
	Highest	23/103 (22.3%)	1.83 (1.12–2.98)	2.30 (1.39–3.79)	2.22 (1.34–3.68)
	*P for trend*		*0.0745*	*0.7151*	*0.5603*

Multivariate model 1: age, sex, stress (high, middle, low), obesity (underweight, normal, overweight), city (metropolitan, middle-small city, rural), academic achievement (high, middle, low), school type (single sex, co-education with same class, co-education with separate class by sex), regular physical activity (≥30 min, 3days and more/week) (yes, no), and 2 hr screen times in usual days (usually, sometimes, never).

Multivariate model 2: Multivariate model 1+ perceivedweight status(thin, normal, fat).

## Discussion

This nationwide cross-sectional online survey of Korean adolescents found an overall prevalence of DEB of 12.7%; the prevalence was higher among girls (14.8%) than among boys (10.5%). U-shaped and J-shaped associations between SES and DEB were observed in Korean girls and boys, respectively.

Studies using the EAT-26 with non-clinical samples found rates of DEB that were higher than or similar to those found in this study [7], [8]. One study reported that the prevalence of DEB was 9.7% among 1,829 girls aged 12–18 years in Canada [8], and another study found the prevalence of DEB to be 22% in girls and 2.0% in boys in a sample of 666 students in the US [7]. A more recent national study conducted in US high schools found that the prevalence of ED was 14.4% among girls and 3.8% among boys in a sample of 5,618 students [9]. One recent Asian study found that the prevalence of DEB was 10.4% among 835 Taiwanese females, [2] and another found a prevalence of 3.9% among boys and 6.5% among girls in a sample of 2,382 secondary school students in Hong Kong [3]. However, it is hard to compare these rates with those observed in the present study because the previous Asian studies were not conducted on a nationwide sample. According to Makino’s review [6], which considered various studies using different measures of EDs, the prevalence of EDs in non-Western countries appears to be increasing, and those with DEB during adolescence were at increased risk for EDs 10 years later [14]. Moreover, EDs during adolescence may be associated with elevated risk for a broad range of physical and mental health problems in adulthood [34].

In addition, the prevalence of DEB in boys was high compared to the other study mentioned above. A review suggested adolescent boys with an eating disorders tend to be overweight, have body dissatisfaction (e.g. want to lose body fat, develop a leaner and more muscular body), and may be affected by gay culture’s increased emphasis on physical appearance [35]. The pressure to be thin in Korea may affect boys’ eating behaviors because male K-pop stars are very slim and youthful. We assumed that appearance became a more important issue to boys. It is warranted for future research to study what exactly was associated with the risk of boys in detail.

Previous community- or school-based studies have reported diverse results about the association between SES and EDs [18], [20], [25], [26], [36]–[40]. Deleel’s study [37] found no significant differences in DEB across SES groups in a sample of 518 young females. Other studies have found an increased risk of ED in upper-SES individuals [16], [20], [22]. On the other hand, several studies [25], [26] found that disordered eating was more prevalent in the lower SES group.

Most recently, a national study among Mexican adolescents found that higher SES was associated with higher risk of DEB [18]. However, our study found that the prevalence of DEB was elevated not only in the highest SES but also in the lowest SES group. A study conducted in one province in South Korea also found that both high and low SES were associated with DEB in girls in the fourth and seventh grades [32].

We can provide no clear explanation for the U- and J-shaped associations revealed in our results. We observed similar findings in a 2 year follow-up survey as in this study. One possible explanation is that the higher ED risk among those with the highest SES may be due to perfectionism [41]. High academic achievement among those in the highest SES group in our study may support this hypothesis. However, this explanation may not be applicable to the lowest SES group in light of the lower academic achievement of those in this group. Given the high prevalence of overweight individuals and those with negative body images in the lowest SES group, we considered that these factors may be connected to higher risk for EDs [42]–[45]. These patterns were much stronger in girls than in boys. Although these explanations of our results may be plausible, other factors may be involved, and interactions among these factors may exist. Further studies are needed to examine the U-shaped association more clearly.

Our study has several strengths. This is the first nationwide survey on EDs in Korean adolescents. We also used the EAT-26, a highly sensitive and reasonably specific instrument with the ability to identify subjects at high risk for ED and to evaluate the risk for ED [30], [46].

This study has also several limitations. First, SES levels were determined from perceived self- reported data. The correlation between income level reported by parents and self-reported SES level reported by adolescent subjects was moderate (r = 0.6, p<0.01) in the sub-sample (n = 50). It was hard to get information concerning household income through asking their parents because of budget problems. The determination and inclusion of more exact household income data is warranted for future studies in order to confirm the results of our study. Second, our cross-sectional study as a baseline for OnKAP survey was not optimal for the investigation of the direction of casual inferences. Third, our study population was drawn from online panels, and data was collected via questionnaires. Respondents in online survey may be more conscious about health and eating disturbance issues, hence the prevalence may be overestimated. However, participants may be more comfortable using an online as opposed to an in-person reporting process. Although almost all Korean middle and high school students use the Internet, non-panel students may differ from panel participants due to the potential selection bias of an on-line panel [47], [48]. Although we used a validated Korean version of the EAT-26, such bias might have led us to miscalculate the number of young people at risk. Furthermore, an accurate diagnosis would require a clinical evaluation [49]. This study suggests that both the lowest and the highest SES are associated with increased risk for developing an ED and that these patterns are stronger in girls. Further research is necessary to understand why the girls at the extremes of SES display such behavior. Most importantly, more public attention should be devoted to adolescents in both the lowest and the highest SES groups to identify and manage EDs. Based on this outcome, public programs for preventing or treating of ED should focus on controlling obesity as well as correcting perceived weight status for the lower SES groups meanwhile focusing on correction of perceived weight status for the higher SES groups.

## Supporting Information

Table S1 **The correlation between adolescents-reported household economic status and parents-reported annual household income in sub-samples (n = 50).** ^1^ 1: lowest 1, 2: low, 3: middle, 4: high, 5: highest. ^2^ million Korean won per year.
(DOCX)
