# Supplementary material for: The Association between Disturbed Eating Behavior and Socioeconomic Status: The Online Korean Adolescent Panel Survey (OnKAPS)
Source: PLoS One. 2013 Mar 5;8(3):e57880. doi: 10.1371/journal.pone.0057880 (PMC3589486; doi:10.1371/journal.pone.0057880)
Supplement: Table S1 — The correlation between adolescents-reported household economic status and parents-reported annual household income in sub-samples (n = 50). 1 1: lowest 1, 2: low, 3: middle, 4: high, 5: highest. 2 million Korean won per year. (DOCX) [file pone.0057880.s001.docx]

Supplementary table 1. The correlation between adolescents-reported household economic status and parents-reported annual household income in sub-samples (n=50).

| SES | Mean | Standard deviation | Min | Max | Correlation |
| --- | --- | --- | --- | --- | --- |
| Adolescents-reported household economic status^1^ | 2.9 | 0.91 | 1 | 5 | R=0.64  P<.0001 |
| Parents-reported annual household income by phone survey^2^ | 5.02 | 1.88 | 2.2 | 8.7 |  |

^1^ 1: lowest 1, 2: low, 3: middle, 4: high, 5: highest

^2^ million Korean won per year
